# Difference and Variance in Nutrient Intake by Age for Older Adults Living Alone in Japan: Comparison of Dietary Reference Intakes for the Japanese Population

**DOI:** 10.3390/nu13051431

**Published:** 2021-04-23

**Authors:** Midori Ishikawa, Hiroshi Yokomichi, Tetsuji Yokoyama

**Affiliations:** 1 Department of Health Promotion, National Institute of Public Health, 2-3-6 Minami, Wako, Saitama 351-0197, Japan; yokoyama.t.aa@niph.go.jp; 2 Department of Health Sciences, School of Medicine, University of Yamanashi, 1110, Shimokato, Chuo, Yamanashi 409-3898, Japan; hyokomichi@yamanashi.ac.jp

**Keywords:** older adults, usual intake of nutrients, within- and between-individual variances, dietary reference intakes for the Japanese population, AGEVAR MODE, difference by age

## Abstract

This study aimed to estimate the distribution of usual intakes in protein, sodium, potassium, and calcium by age group and assessed whether proportions of deficiencies/excesses of each nutrient would occur more in older age via a comparison with the dietary reference intakes for the Japanese population (DRIs_J). A cross-sectional analysis was conducted using a database of the 2-day nutrient intake of 361 Japanese people aged 65–90 years. The AGEVAR MODE was used to estimate usual intake. Percentile curves using estimated distribution by sex and age and usual nutrient intake were compared to those of the DRIs_J. The usual intake of protein (male and female) and potassium and calcium (female) were lower with older age. Within-individual variance of protein in female (*p* = 0.037) and calcium in male (*p* = 0.008) subjects were considerably lower with older age. The proportion of deficiencies in protein (male and female), potassium (female), and calcium (female) were higher with older age. However, the proportion of people with excess salt (converted from sodium; male and female) did not differ by age. The variances found herein could be important for enhancing the understanding of differences in dietary intake by age.

## 1. Introduction

In older adults, physiological problems related to aging, such as motor and cognitive functions, influence dietary intake among individuals. These problems also increase individual differences in the usual intake of nutrients by age and can lead to frailty [1,2].

A previous study, based on a comparison between 30–49- and 50–69-year-old females and between 30–49- and 50–76-year-old males, reported that the within- and between-individual variances of dietary intake differed by sex, age, and nutrients [3].

However, there are only a few studies that identify the differences in nutrient intake by age groups, particularly among older adults. Owing to the small sample size for each age group [4], the standard error of the differences in nutrient intake by age and inter-individual variance would be large, which has made the measurement of differences difficult [5].

The AGEVAR MODE [6] method is a model that can express the average value of nutrient intake and within- and between-individual variances by age. The method helped overcome these limitations and demonstrated smaller standard errors compared with the other models and less bias despite small sample sizes using data from dietary surveys that lasted for a minimum of 2 days.

The current study used the AGEVAR MODE method and assessed differences in usual nutrient intake and within- and between-individual variances of important nutrients, such as protein, sodium (converted to salt equivalent), potassium, and calcium for older adults by age. In addition, data were compared with DRIs_J, and the differences in the proportion of individuals with deficiencies in or excess of nutrients by age were identified.

The study hypothesized that old-old adults (75 years and more) would have higher deficiencies of protein, potassium, and calcium, and excesses of sodium, compared with young-old adults (65–74 year). It has been reported that old-old adults prefer the traditional Japanese diet that contain a high amount of salt [7].

The proportion of older adults living alone has increased in Japan. Therefore, this study may contribute to the assessment of recommended nutrient intake and appropriate countermeasures for older adults.

## 2. Materials and Methods

A cross-sectional analysis was conducted using the existing database of a cross-sectional nationwide survey in Japan. The original study aimed to clarify the relationship between health and nutritional status, diet, geographical environment, and food access of older adults living alone, which was conducted in 2013 and 2014. The relationships between health and physical status [8,9], food accessibility [10], and eating behaviors [11] as well as the distribution of the usual intake of nutrients were reported. However, differences in nutrient intake by age were not identified [8].

### 2.1. Study Population and Procedure

Figure S1 provides the study population and flowchart of the study.

First, the researchers sought research approval from the mayor of each town or city. The subjects were recruited according to geographical factors through municipal support. Data were obtained from the basic residents’ register on older citizens (individuals aged between 65 and 90 years) who lived alone. Then, distance from the home of each eligible respondent home to the nearest supermarket (by road) was evaluated and mapped using a geographic information system (ArcGIS 10.2; ESRI Inc., Redlands, CA, USA). Subsequently, a list of supermarkets and their geographic locations was obtained from a telephone directory published in October 2010 (Telepoint Pack!; Zenrin Inc., Tokyo, Japan). Based on previous studies [10], potential respondents were categorized into the following groups: (a) those living within 500 m, (b) between 500 m and l km, (c) and >1 km away from the nearest supermarket. An equal number of prospective participants were selected in a random stratified manner from the urban central, rural, and mountainous areas of each city or town. In total, 534 participants responded to the 2-day weighed dietary record survey. The researchers obtained written informed consent from all participants. The study excluded respondents who failed to answer the items necessary for analysis and derived data from 109 male and 252 female participants. The participants answered all items, including nutrient intake, nutritional status, frailty, and other variables presented in the self-administered questionnaire.

### 2.2. Nutrient Intake by 2-Day Weighed Dietary Records

Dietary records were obtained using the methodology of the National Health and Nutrition Survey in Japan (NHNS_J) [12]. To calculate highly reliable nutrient intake from a 2-day dietary record, trained registered dietitians conducted the survey from October 2012 to October 2013. Each participant received two recording papers and instructions on how to record information. The participants weighed the food consumed and enumerated each dish and ingredient and the quantity consumed. The registered dietitians or trained staff visited the homes of all participants at least once during the survey and confirmed and completed the dietary records through interviews [8]. Using the nutrition analysis software Excel Eiyoukun version 8.0 (corresponding to Standard Tables of Food Composition in Japan 2015), energy and nutrient intake were evaluated according to the 2015 Standard Tables of Food Composition in Japan [13]. Sodium was converted into salt equivalent for comparison with DRI_Js.

The study presents the results for protein, salt equivalent, potassium, and calcium. These nutrients were selected from the perspective of hypertension, osteoporosis, and frailty, which are serious health problems concerning Japanese older adults. Specifically, salt and potassium are associated with hypertension; calcium and protein are associated with osteoporosis and frailty, respectively.

### 2.3. Usual Nutrient Intake Estimated Using the Agevar Mode

Dietary surveys require 3–7 days to estimate usual intake. However, the study refrained from following the typical timeframe because it tends to increase the burden on the target older person [8]. Therefore, the researchers reviewed several methods that estimate the distribution of usual nutrient intake in a given population by conducting a multi-day dietary survey [14]. Many of such recognized methods are of the National Research Council/Institute of Medicine [15], Iowa State University [16], and the National Cancer Institute [17]. Yokomichi and Yokoyama, members of the present study, proposed a mixed-effect model named the AGEVAR MODE with age-dependent mean and within- and between-variances. The AGEVAR MODE method is a model that can express the average value of nutrient intake and within- and between- variances by age [6]. Furthermore, the method demonstrated smaller standard errors, especially for age-dependent analyses, compared to other models and less biases even for small sample sizes using data from a minimum of 2 days of dietary surveys [6]. Therefore, the method was considered appropriate for the study in the estimation of usual intake by age.

### 2.4. Body Measurements

The height measurements of the participants were derived from their most recent health examination. Height and weight were measured once before the morning meal. In the case of absence of information, the researchers measured it using a portable measuring device. Weight was measured using a digital scale. Body mass index (BMI) was evaluated for each participant as weight (kg) divided by height (m^2^).

### 2.5. Frailty and Other Variables

A self-administered questionnaire was used for identifying the characteristics of older adults by collecting data on socioeconomic factors, such as age, sex, annual income, highest level of education attained, special nursing needs, and diseases. The frailty index was then measured using a previously developed method used by the Tokyo Metropolitan Institute. The index was a validated checklist for preventive care and used for screening older Japanese people for frailty [18].

The checklist comprises 15 self-administered items, such as being homebound; communication with friends, neighbors, and family members or relatives; physical conditions, such as capability to walk continuously for 1 km; comfortable vision; chewing ability; nutritional status; and a history of falls. The index score ranges from 0 to 15 with a high score suggesting high levels of frailty. Respondents with a score of 4 were classified as frail [18]. Lastly, the requirement for long-term nursing care was assessed.

### 2.6. Statistics

Data on 109 male and 252 female adults who answered all necessary items for the study were analyzed. The distribution of the usual intake for each sex and age class of older adults was calculated by following steps 1–7 listed below as per the AGEVAR MODE [6]. Furthermore, the proportion of usual nutrient intake was estimated under the estimated average requirement (EAR) or tentative dietary goals (DGs) for preventing lifestyle-related diseases of DRIs_J for each sex and age class by following steps 8–9 listed below:

1. Amounts of daily nutrient intake were surveyed for 2 days to be approximately normally distributed using the Box–Cox transformation [19].
2. The mean of the transformed data was modeled to be explained by the polynomial and logarithmic functions of age and within- and between-individual variances of data using a monotone exponential function of age. In this step, the transformed scale of the nutrient was assumed. Moreover, within- and between-individual variances of daily nutritional intakes should be normally distributed around the estimated mean of each individual and at each age group, respectively.
3. Within-individual variance was then omitted from the estimated nutrient distribution for each age group given that the estimated mean and between-individual variance would establish the distribution of usual nutritional intake in the transformed scale.
4. For the estimated distribution of usual nutritional intake in the transformed scale, percentiles of the data were estimated using the mean and between-individual variance for each age group.
5. The distribution of the usual nutrient intake in the original scale of the nutrients was obtained by a reverse transformation of the usual intake distribution in the Box–Cox transformed scale. In this step, the estimated percentiles were reversed.
6. Previous studies reported that bias would be mathematically induced in reverse transformation [14]. Therefore, the bias was reduced as per the equation, and the final distribution of usual intake in the original scale was obtained. In other words, within- and between-individual variations reported in the study are those observed before eliminating within-person variance to estimate usual intake.
7. The estimated distribution was then graphically presented as percentile curves representing usual nutrient and daily intakes in the original and transformed scales, respectively, and curves representing within- and between-individual variances and the ratio of within-/between-individual variance in the transformed scale. The shaded areas of the 2.5%, 50%, and 97.5% percentile curves indicate the standard errors of the estimated intake distribution to demonstrate the magnitude of the estimation error owing to the sample size.
8. Furthermore, the usual intake of nutrients was assessed by comparing the current observations with the 2020 DRIs_J. The survey was conducted between 2012 and 2013. However, the DRIs_J version was used for analysis because it was developed on the basis of new evidences and was a standard that is comprehensively divided into periods for older adults. For the 2020 DRIs_J, the reference intake is set for the age groups of 65–74 and ≥75 years [20]. The DRIs_J for the EAR for protein (g/day) indicated that men and women aged 65–74 years and 75 years and above require ≥50 and ≥40 g/day of protein, respectively. For DGs (tentative dietary goals for preventing lifestyle related diseases) of salt equivalent (g/day), the DRIs_J imply that men and women aged 65–74 years and 75 years and above need to consume <7.5 and <6.5 g/day, respectively. For the DG of potassium (mg/day), the DRIs_J suggest that men and women aged 65–74 years and 75 years and above should consume ≥3000 and ≥2600 mg/day, respectively. In terms of the EAR of calcium (mg/day), ≥600 and ≥550 mg/day are appropriate for men and women aged 65–74 years and 75 years and above, respectively.
9. The graph displays changes with age of the estimated percentage of individuals under the EAR or those who exceeded the DG in DRIs_J.
10. Statistical analyses were performed using SAS software version 9.4 (SAS Institute, Inc., Cary, NC, USA). A *p*-value of <0.05 was considered statistically significant.

## 3. Results

Table 1 lists the characteristics of young-old (aged 65–74 years) and old-old (aged 75 years or more) adults. In males, a correlation was not observed between age category (young-old and old-old) and residential area, disease (not shown in the table), and frailty scores. The annual income for old-old males was significantly higher than that for young-old males (*p* = 0.007). Although many old-old males were elementary school graduates, several young-old males had attained university education (*p* = 0.001). Moreover, among the young-old males, the nutritional status based on height, weight, and BMI tended to be lower than that for old-old males. In females, no relationship was observed between age category and residential area. However, the characteristics of annual income, highest educational qualification, nutritional status, disease, nursing care needs, and frailty status among old-old females were significantly worse than those for young-old females. In addition, the proportion of old-old females with a frailty score of >4 points was significantly higher than that of young-old females (*p* = 0.0001).

**Figure 1:**
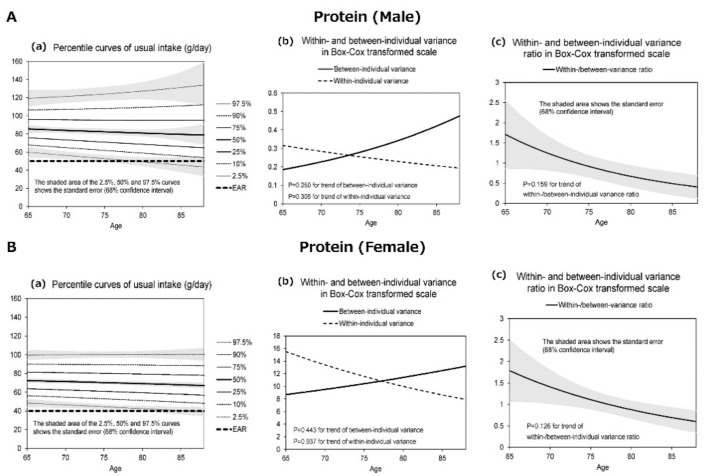
The percentile curves of usual nutrient intake of protein by age (male and female).

**Figure 2:**
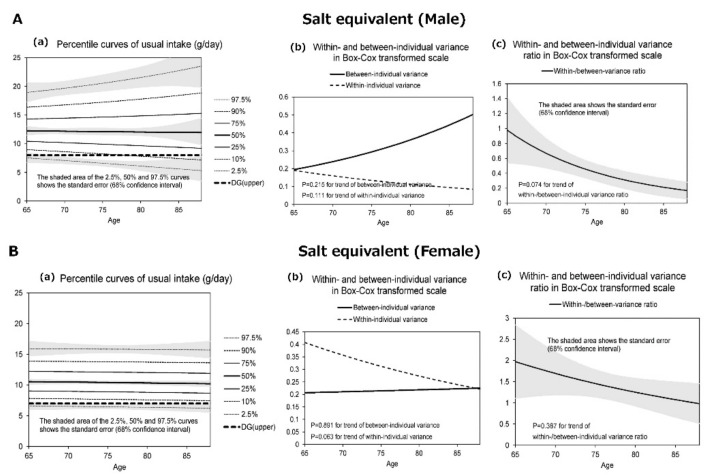
The percentile curves of usual nutrient intake of salt equivalent by age (male and female).

**Figure 3:**
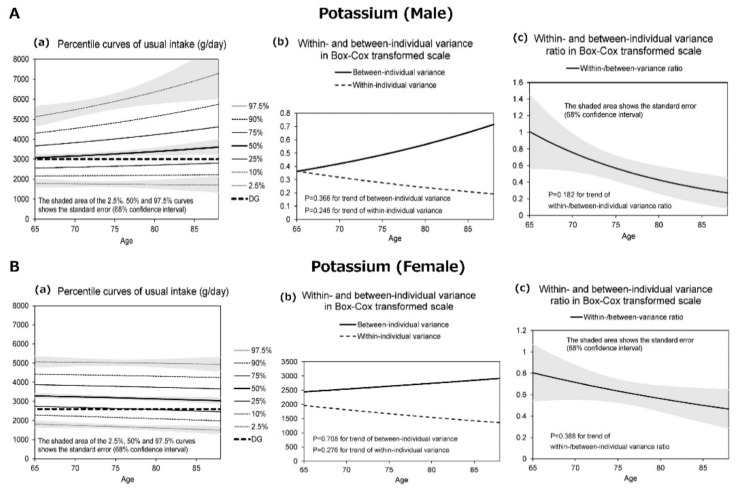
The percentile curves of usual nutrient intake of potassium by age (male and female).

**Figure 4:**
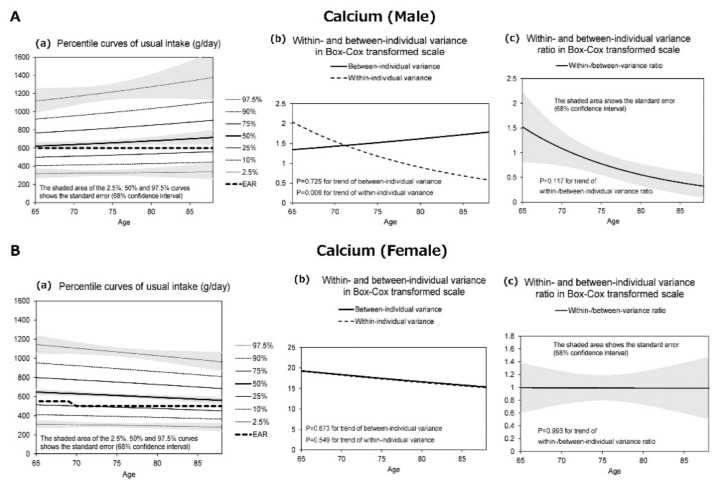
The percentile curves of usual nutrient intake of calcium by age (male and female).

Figure 1, Figure 2, Figure 3 and Figure 4 display the results for protein (Figure 1), salt equivalent (Figure 2), potassium (Figure 3), and calcium intake (Figure 4) as important nutrients for older male and female adults.

In addition, (a) presents the diagrams for the percentile curves of usual intake (g/day), (b) within- and between-individual variances, and (c) their ratios in the Box–Cox transformed scale. The percentile curves (a) (i.e., 2.5%, 10%, 25%, 50%, 75%, 90%, and 97.5%) of intake for each nutrient are presented separately for males and females. The shaded areas of the 2.5%, 50%, and 97.5% curves indicate the standard error that corresponds with 68% confidence interval, whereas the bold dotted line presents the 2020 DRIs_J values.

The results revealed that the usual intakes of protein (male and female), salt (male), potassium (female), and calcium (female) were lower with the increase in age. However, the within- and between-individual variances of each nutrient differed according to sex and age.

### 3.1. Usual Nutrient Intake

Proteins were selected as indicators of muscle weakness in undernutrition. Salt and potassium were selected as indicators of hypertension, which is the main nutritional problem affecting the Japanese population. Finally, calcium was selected as an indicator of osteoporosis.

Figure 1A(a) illustrates the differences in the percentile curves of the usual intake for proteins by age in males. The protein intake decreased with increasing age. The proportion of individuals who ingested less than the EAR (50 g) of the DRIs_J was low and marginally observed in those aged 80 years or more. Figure 1B(a) illustrates the differences in the percentile curves of usual intake for proteins by age in females. The usual protein intake decreased with an increase in age. However, a few women ingested less than the EAR (40 g) even in their old age.

Figure 2A(a) illustrates the differences of the percentile curves for the usual intake of salt by age in males. The median salt intake was nearly constant at high levels (~10 g) but was distributed according to increases in age. Therefore, >80% of the participants at all age groups consumed more than the DG (7.5 g). Figure 2B(a) shows that approximately 90% of the female participants ingested salt beyond the upper limit (6.5 g) of the DG at all groups age. In addition, the between-individual variance of salt intake for females did not exhibit any substantial changes by age (Figure 2B(b)).

Figure 3A(a) demonstrates changes in the percentile curves for the usual intake of potassium by age in males. The median potassium intake was higher, whereas the percentile curves were distributed with the increase in age. Consequently, the proportion of participants who consumed less than the DG (3000 mg) was greater than 25% at all age groups. In Figure 3B(a), the usual intake among females decreased with the increase in age. Therefore, the proportion that ingested potassium below the DG (2600 mg) slightly increased and reached ~25% at 85 years.

Finally, Figure 4A(a) shows the differences in the percentile curves for the usual intake of calcium by age in males. Calcium intake increased with an increase in age. At 65 years, the proportion who consumed less than the EAR (600 mg) was ~50%. However, this rate decreased with an increase in age. In Figure 4B(a), calcium intake was lower among females, and it gradually increased with an increase in age. At 65 years, the proportion who ingested less than the EAR (550 mg) was ~25%. However, because the EAR for individuals aged 75 years or more was set to low (500 mg), the proportion with less than the required amount did not differ considerably.

### 3.2. Within- and between-Individual Variances of Nutrients

Figure 1, Figure 2, Figure 3 and Figure 4 further display the within- and between-individual variances of nutrient intake. For males and females, several nutrients existed with large within-individual variances compared to between-individual variances. In particular, the within-individual variance in protein for females (*p* = 0.037) (Figure 1B(b)), salt for females (*p* = 0.063) (Figure 2B(b)), and calcium for males (*p* = 0.008) (Figure 4A(b)) decreased with the increase in age. In other words, the day-to-day variations in the intakes of these nutrients were lower for old-old adults. Furthermore, with regard to protein (male and female) and calcium (male) intakes, the between-individual variance until the age of 70–75 years was less than within-individual variance. However, at more than 70–75 years, between-individual variance was higher than within-individual variance.

### 3.3. Proportion of Individuals with a Usual Intake Less or More Than the Estimated Average Requirement (EAR) or Tentative Dietary Goals for Preventing Lifestyle-Related Diseases

**Figure 5:**
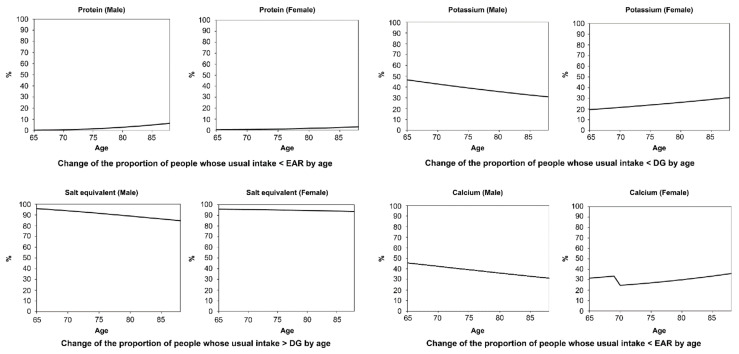
The proportion of individuals whose usual intake was under the estimated average requirement (EAR) or dietary goal (DG) in protein, salt equivalent, potassium and calcium (male and female).

Figure 5 presents the proportion of individuals with a usual intake less than the EAR or more than the DG for protein, salt equivalent, potassium, and calcium. The proportion of individuals with deficiencies in protein (male and female), potassium (female), and calcium (female) decreased with the increase in age. However, the proportion of individuals with excess salt (male and female) did not differ much by age.

## 4. Discussion

The study estimated the usual intakes of protein, sodium, potassium, and calcium by age group, and the deficiencies/excesses in intake proportions were assessed by comparing the results to the Dietary Reference Intakes for Japanese. The results confirmed the differences in the usual intakes of nutrients by age. Among the nutrients, protein, which is one of the vital nutrients required for older adults, exhibited a decrease with the increase in age. Individuals whose intake was under the EAR appeared at age 70–75 years, whereas the percentage of those with consumption under the EAR decreased with the increase in age (male and female). A previous study proposed the association between aging, frailty, and protein intake, including total protein [21].

The within-individual variance of protein for the female participants was remarkably reduced with the increase in age. However, the proportion of old-old adults who consumed less than the EAR was low. Many studies have confirmed low levels of food diversity among males, and males and females may have lesser meat intake with aging [8,22]. In future, investigating the relationship between nutrients and food types is imperative [23] with the coding of food groups as a background [24].

With regard to salt and potassium intakes for females, no difference was observed in salt intake. However, potassium intake decreased with the increase in age. This finding could be attributed to concentrated seasoning. In the study, 56% of the females were hypertensive, which was consistent with the results of a previous study conducted by the researchers [8] and another study on elderly Chinese individuals [25]. In addition, Villela et al. [26] reported that older adults with hypertension prefer salty foods more than those without hypertension. Moreover, in old-old adults, seeking methods to support dietary intake is necessary.

The usual calcium intake among males increased with the increase in age. One of the reasons for this result is that the target population comprised senior adults who lived alone. That is, they could live autonomously without availing of elderly care facilities because these individuals had a nutrient intake more than the EAR. Such individuals are likely to be careful about their calcium intake. The proportion of individuals below the EAR decreased with the increase in age. The studies conducted in China and Germany reported similar results. In other words, the dietary intake of senior adults living independently, including super-aged individuals, was adequate for the majority of the evaluated nutrients [25,27].

The features of nutritional status in the older population emerged clearly in terms of percentiles for usual intake as well as within- and between-individual variances by applying the AGEVAR MODE to the 2-day dietary survey data. The variance of 1-day dietary intakes in the population comprised between- and within-individual variances [5,28]. Many studies encouraged the discontinuous 2-day surveys for short-term dietary surveys to estimate the usual nutrient intake [29]. Using the AGEVAR MODE, the present study indicated the feasibility and applicability of using non-consecutive 2-day surveys of older adults to assess usual dietary intake. In fact, depending on the nutrients, the usual intake variance and within- and between-individual variances differed [30]. Therefore, the AGEVAR MODE successfully estimated the percentiles for usual nutrient intakes and within- and between-individual variances of interest and enabled the assessment of differences in nutritional status by age in an elderly population. However, the model can only address dietary data that are normally distributed with appropriate transformation and, therefore, may require further improvement, such that it could be successfully applied to data with large numbers of null amount or data of food items [31]. Although the AGEVAR MODE yields smaller standard errors of age-dependent estimates compared with the other models [6], the sample size of the current study may not be enough especially in males. To consider this issue, we conducted post hoc power analyses to detect a significant (*p* < 0.05) change in within- and between-individual variances and their ratio according to age, given the effect size (the percent change per 10-year increment of age) observed in the current study. Among the 4 nutrients (protein, salt equivalent, potassium, and calcium), the median effect-size of within- and between-individual variances and their ratio (and post hoc power) was −27% (0.30), +42% (0.18), and +91% (0.32) in men, respectively; −19% (0.30), +6% (0.04), and +31% (0.13) in women, respectively. Thus, the non-significant changes according to age may be due to the small sample size in men and the small effect size in women.

Although the model corresponds to various median amounts of usual intake, its estimation is based on the assumption that within- and between-individual variances would either monotonously be high or low. The study assumes that this assumption would be natural in real-world settings because a previous study suggested that older adults tend to have low within-individual variance and high between-individual variance with the increase in age [6,25]. However, the variances may change over time among young and middle-aged adults, a subset of whose dietary lives would drastically change [32]. They may live alone far from their hometown for business reasons and would marry and raise children. A subset may even obtain a divorce. The considerations of employment status and family member data may be necessary for future studies on improving such statistical models to estimate the distribution of usual intake.

The present study has certain limitations and requires improvements in the future. First, the researchers omitted weekends or holidays in the dietary survey because many of the subjects were retired. However, their dietary behavior and meals during weekends may differ during weekdays because meals would be shared with relatives, children, or grandchildren. In addition, the survey was conducted between October and November, which is the autumn season. Further studies should include food type and nutrients of food with seasonal effects and changes [33]. Second, the participants included only those who were living alone. In this regard, comparing the results between older adults who live alone and those who live with others is necessary. Third, the number of male participants was less than that of female participants in the survey. The researchers faced difficulties in obtaining consent for study cooperation from male subjects. In addition, this study analyzed the data of 361 individuals (males = 109; females = 252; 173 persons were excluded) and identified significant differences between the characteristics of the included versus the excluded participants. The results indicated that among the excluded participants, the proportion of individuals who required special nursing care was significantly higher than those who did not. The study considered the proportion small to influence the results because it targeted individuals who could eat independently. Fourth, future studies should consider that the target population in the central area is lesser than the real population because equal numbers of prospective participants were selected on the basis of the distance from the home to the supermarket. Fifth, the study was a cross-sectional analysis, which could not examine changes over time. Further research on changes over time is required in the future.

Despite these limitations, the study demonstrated differences in the usual intake and within- and between-individual variances of nutrients according to the age of older adults living alone.

The proportion of old-old adults living alone has increased in Japan. Therefore, this study contributes to the assessment of the recommended nutrient intake and appropriate countermeasures for older adults taking consideration of aging.

## Figures and Tables

**Table 1:** Characteristics of the young-old (65–74 years) and old-old (75–90 years) adults.

		Age (Years)									
		Male					Female				
		65–74 Years (*n* = 64)		75–90 Years (*n* = 45)		*p*	65–74 Years (*n* = 128)		75–90 Years (*n* = 124)		*p*
		*n*	%	*n*	%		*n*	%	*n*	%	
Annual income (JPY) ^1,2^	≥4 million (≥$36,364)	4	3.1	4	4.4	0.007	4	1.6	6	2.4	<0.0001
	2–4 million ($18,182–36,363)	46	35.9	48	53.3		62	24.2	54	21.8	
	1.5–2 million ($13,636–18,181)	36	28.1	10	11.1		64	25.0	26	10.5	
	1–1.5 million ($9090–13,635)	16	12.5	18	20.0		66	25.8	52	21.0	
	<1 million (<9090)	10	7.8	4	4.4		38	14.8	78	31.5	
	do not want to answer	16	12.5	6	6.7		22	8.6	32	12.9	
Highest level of education attained ^2^	University graduate school	30	23.4	28	31.1	0.001	8	3.2	4	1.6	0.004
	College, special school	16	12.5	2	2.2		38	15.0	24	9.7	
	High school	62	48.4	30	33.3		108	45.5	82	33.1	
	Primary, secondary school	18	14.1	26	28.9		90	35.4	130	52.4	
	Do not want to answer	2	1.6	4	4.4		10	3.9	8	3.2	
Nutritional status ^3^	Height (mean; SD)	164.0	7.6	161.8	5.5	0.015	150.4	5.3	147.9	5.6	<0.0001
	(minimum–maximum)	133	178	148	169		135	164	130	158	
	Weight (mean; SD)	63.9	10.2	59.8	8.6	0.007	52.9	8.1	49.2	7.1	<0.0001
	(minimum–maximum)	45	93	40	78		31	76	32	73	
	BMI (mean; SD)	23.7	2.9	22.9	3.1	0.064	23.4	3.3	22.5	3.1	0.001
	(minimum–maximum)	16.9	32.3	16.4	29.5		15.4	32.9	15.6	32.8	
Special nursing needs ^2^	No	126	98.4	84	93.3	0.048	254	99.22	220	88.71	<0.0001
	Yes	2	1.6	6	6.7		2	0.78	28	11.29	
Frailty index (Kaigo–Yobo checklist) ^2^ *(0–15 points)*	≥4 points	22	17.2	24	26.7	0.091	30	11.7	78	31.5	<0.0001
	<4 points	106	82.8	66	73.3		226	88.3	170	68.6	

^1^ 1$ = 110 yen, June 2020–present; ^2^ χ^2^ test or Fisher’s exact test; ^3^ *t*-test.

## Data Availability

The datasets used and/or analyzed during the current study are available from the corresponding author on reasonable request.

## Supplementary Materials

The following are available online at https://www.mdpi.com/article/10.3390/nu13051431/s1. Figure S1: Study population and flowchart of the study.
