# Supplementary material for: Difference and Variance in Nutrient Intake by Age for Older Adults Living Alone in Japan: Comparison of Dietary Reference Intakes for the Japanese Population
Source: Nutrients. 2021 Apr 23;13(5):1431. doi: 10.3390/nu13051431 (PMC8146330; doi:10.3390/nu13051431)
Supplement: Supplementary file 1 [file nutrients-13-01431-s001.zip › nutrients-1147337-supplementary.pdf]

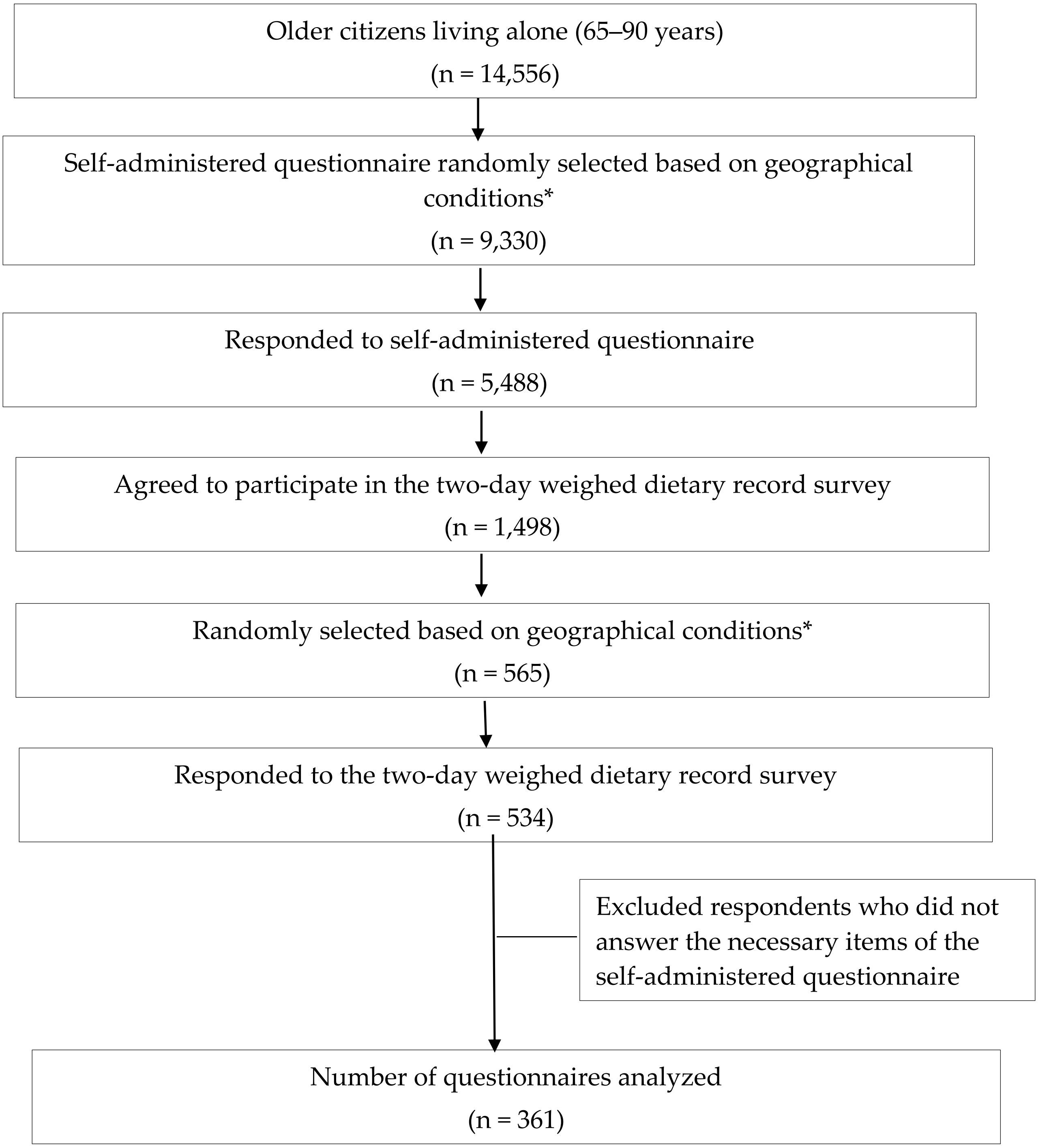

Figure S1. Study population and flowchart of the study.

* A research agreement was exchanged between the researcher dealing with geographic information and mayors in the local government. The research agreement stipulated that the target persons would be identified by the local government in accordance with the rules of confidentiality obligation. Participation in the survey was requested from those selected by the local governments.
